# The Confounding Effect of Population Structure on Bayesian Skyline Plot Inferences of Demographic History

**DOI:** 10.1371/journal.pone.0062992

**Published:** 2013-05-07

**Authors:** Rasmus Heller, Lounes Chikhi, Hans Redlef Siegismund

**Affiliations:** 1 Instituto Gulbenkian de Ciência, Oeiras, Portugal; 2 Department of Biology, University of Copenhagen, Copenhagen, Denmark; Aarhus University, Denmark

## Abstract

Many coalescent-based methods aiming to infer the demographic history of populations assume a single, isolated and panmictic population (i.e. a Wright-Fisher model). While this assumption may be reasonable under many conditions, several recent studies have shown that the results can be misleading when it is violated. Among the most widely applied demographic inference methods are Bayesian skyline plots (BSPs), which are used across a range of biological fields. Violations of the panmixia assumption are to be expected in many biological systems, but the consequences for skyline plot inferences have so far not been addressed and quantified. We simulated DNA sequence data under a variety of scenarios involving structured populations with variable levels of gene flow and analysed them using BSPs as implemented in the software package BEAST. Results revealed that BSPs can show false signals of population decline under biologically plausible combinations of population structure and sampling strategy, suggesting that the interpretation of several previous studies may need to be re-evaluated. We found that a balanced sampling strategy whereby samples are distributed on several populations provides the best scheme for inferring demographic change over a typical time scale. Analyses of data from a structured African buffalo population demonstrate how BSP results can be strengthened by simulations. We recommend that sample selection should be carefully considered in relation to population structure previous to BSP analyses, and that alternative scenarios should be evaluated when interpreting signals of population size change.

## Introduction

Coalescent-based methods can be used to infer demographic change (used here in the narrow sense of population size change) from genetic data [1], [2]. The coalescent framework has contributed important information about the demographic history of humans [3]–[5] and other species [6]–[8]. This has improved our understanding of the factors that have affected past ecosystems, whether climatic or anthropogenic, recent or ancient. Demographic inference methods based on the coalescent usually assume panmixia, i.e. the absence of population structure, although this is not a realistic assumption in many biological situations. A number of recent studies have investigated the effect of violating the panmixia assumption for inferring population size changes [9]–[11]. These studies suggest that population structure can lead to erroneous conclusions about demographic changes in a population that in fact has remained stationary through time.

Bayesian skyline plots (BSPs [2]), or derivatives thereof such as the extended Bayesian skyline plot (EBSP [12]), have become increasingly popular for inferring demographic changes using sequence data. A search on the exact term (conducted December 13^th^ 2012) returned 1310 hits in Google Scholar, covering the spectrum of organisms from viruses to large mammals. Skyline plots assume a single panmictic population and use inferred patterns of coalescence to fit a demographic model to a set of sequence data. Although a recent review highlights the danger of violating the panmixia assumption in BSP inference [13], the structure effect on BSPs has not been quantified. Importantly, the confounding structure effect is not a fault of the skyline methods *per se*, but rather a case of fitting a wrong model (panmixia) to the data. As shown by several authors [11], [14]–[16] it is fundamentally due to the fact that genes sampled within one population (or deme) within a set of inter-connected demes (or a structured population) exhibit genealogies that resemble those of panmictic populations that have declined in size. Structured populations have genealogies that differ from panmictic ones in some crucial aspects. In a seminal paper, Wakeley [14] identified two distinct phases when the genealogy of a structured population is considered backwards in time: the recent scattering phase, which lasts until all sampled lineages have coalesced or migrated so that each remaining lineage is in a separate deme. At this point the genealogy enters the collecting phase, where two or more lineages have to migrate to the same deme before coalescence can occur. Pannell [17] showed how the presence of these distinct genealogical phases will cause an apparent decline in estimates of effective population size going from the ancient (collecting phase) to the recent (scattering phase) part of the genealogy. BSPs derive population sizes from inferred genealogies and will consequently be prone to confound the effect of structure with declines in population size. We define this confounding of structure and demographic change as the ‘structure effect’. The structure effect remains under-appreciated in BSP analyses, and consequently there is a danger of deriving erroneous demographic conclusions from BSP analyses of structured populations. Some BSP-based studies do consider and discuss the possible confounding effect of structure (e.g. [7], [21]), but an evaluation of the magnitude of the structure effect and the conditions that are especially prone to it is lacking. The present study is intended to serve this purpose.

This study expands on previous studies evaluating the structure effect. Städler et al. [9] examined the effect of structure on Tajima’s *D* and Fu and Li’s *D* statistics in growing or constant populations, and Chikhi et al. [10] examined the structure effect on inferences based on MSVAR [18], a program using microsatellite data to infer a single change in population size. As mentioned above, Pannell [17] provided some important insights (albeit in a metapopulation framework) into the structure effect on generalized skyline plots. Here, we address specifically the structure effect on BSPs with the aim of evaluating their robustness and power to distinguish true population size changes when the panmixia assumption is violated. Furthermore, we discuss practical issues that should be considered before interpreting the demographic signal in BSPs. Our results show that population structure and the sampling strategies are issues that must be considered, but also that some sampling strategies can minimize the effect of spurious population size inference.

To put our simulation results into perspective, we supplemented them with a case study of mtDNA data from the African buffalo (*Syncerus caffer*) distributed on 34 distinct localities in sub-Saharan Africa [19]. This structural conformation is comparable (in terms of the number of demes) to the simulation scenarios and allowed us to assess the structure effect in a more realistic setting. By including a case based on real data we demonstrate how simulations can complement analyses of real data to validate observed skyline plot results. The risk of a structure effect in an empirical study depends on many factors, hence is difficult to assess without performing simulations that emulate the structural context in which the real data are collected. The inclusion of real data furthermore enabled us to assess the structure effect when historical changes in the structure and population size according to the known history of the buffalo [20], [21] are incorporated. We acknowledge that the complexity underlying any real data set is not reducible to the simulation scenarios. Yet we think that the simulations and the case can illuminate each other: the case illustrates how the simulated results apply to real-world situations and conversely the simulations lends credibility to the results based on real data.

## Materials and Methods

The analyses were divided in two parts: first, we simulated data under an idealised model with a simplified migration pattern connecting the demes. This was done to illustrate the structure effect on BSPs under standardized conditions. We also considered more complex models that–in addition to the idealised structure–involved changes in either population size or structure. Second, we used data from a set of 755 African buffalo D-loop sequences distributed on 34 distinct populations in sub-Saharan Africa to test the structure effect under a data-informed migration matrix and on real sequence data.

### Simulated Scenarios

#### Simulation settings

We used the program Bayesian Serial SimCoal (BSSC [22]) to simulate DNA sequence data under different structural and demographic models. We simulated a 600bp fragment of the mitochondrial D-loop, commonly used in BSP studies due to its high nucleotide diversity. The sequences were set to evolve according to a HKY model with kappa = 50, gamma distributed rate heterogeneity (shape parameter 0.5) and a rate of 32% per million years per bp [6] (note that this rate is subject to estimation uncertainty, but here it serves to provide a conversion between genetic distance and real time), equivalent to 0.001344 mutations per sequence per generation (using the estimated buffalo generation time of 7 years [20]). We emulated the actual marker used in the buffalo case study in the simulations to facilitate comparisons, and because BSPs are almost always used in the context of dated genealogies with time measured in years. For all scenarios, we carried out 100 replicate simulations to incorporate coalescent stochasticity [23] and identify general patterns across stochastic replicates of the same demographic history. Essentially, this corresponds to simulating 100 non-linked genetic markers with the high information content of the D-loop. We were thus able to assess the performance of multi-locus inference and ensure that our conclusions were not limited by the use of a single locus. This makes our results more comparable to multi-locus data that are likely to become common in the genomic era. Two example input files for BSSC are supplied to show the details of our simulations (File S1 and File S2).

#### Sampling strategy

The influence of the sampling scheme was investigated by drawing 40 samples in three different ways: 1) all 40 samples from a single deme, 2) 4 samples from each of 10 demes and 3) one sample from each of the 40 demes in the structured population. These correspond respectively to the *local, pooled* and *scattered* (not to be confused with the term ‘scattering phase’, which refers to aspects of the underlying genealogy) sampling regimes described in Städler et al. [9] and Chikhi et al. [10], and we retain that terminology (always in italics) throughout this study. Unless otherwise stated, these three sampling strategies were explored for all simulated scenarios.

#### Idealised structured population

The most simple simulation scenario was an island model with 40 demes of 500 mtDNA copies (corresponding to 500 females) connected by equal migration and with a stationary population size throughout. Three levels of migration rates were simulated: *N_f_m* = 0.125, *N_f_m* = 1.25 and *N_f_m* = 12.5 (where *N_f_m* is the number of female migrants per generation, since we are concerned with maternally inherited mtDNA sequences throughout). These rates represent the extremes and an intermediary level of gene flow found in structured populations [9], [10]. The corresponding equilibrium *F*_ST_ values (for an mtDNA marker in an infinite island model [24]) are 0.80, 0.28 and 0.04, respectively. We use the number of migrants (*N_f_m*) to denote the level of gene flow, although we point out that *F*_ST_ values are more directly comparable across marker types and inheritance scalars. In keeping with Städler et al. [9] and Chikhi et al. [10], we supplemented the island model with simulations under a stepping-stone model (see Supporting Information S1) using similar parameter values. We also considered a structured population identical to the 40-deme island model, but with only ten demes (with *N_f_ = *2000 in each) to evaluate the structure effect under these conditions that match more closely a typical population genetic study (although the limited number of sampled demes is probably often indicative of logistic or resource limitations rather than the actual number of demes in structured populations).

In addition to the constant-size idealised scenarios, we considered scenarios where population sizes varied, maintaining structure as above. The population size changes we explored were 1) a single transition from a constant to a ten-fold exponentially declining/expanding population at either a Holocene or Pleistocene time point (see Supporting Information S1 regarding the selected time points); 2) a boom-bust (combining the above Pleistocene growth and Holocene decline) demographic history as recently inferred for the African buffalo [21]. In this way, we wanted to test how different demographic changes might be mimicked or concealed by concomitant population structure under different sampling regimes. For all non-constant demographic scenarios, we explored only the intermediate *N_f_m* = 1.25 gene flow level to reduce computation time and because we already demonstrated the effect of varying gene flow. Finally, we simulated date under an ‘IM-like’ model where structure was not permanent (Supporting Information S1).

#### Data-informed structured population

To assess the structure effect under more complex conditions than the idealised, equal-migration scenarios mentioned above, we carried out simulations that mimicked the structure in a data set consisting of 755 D-loop sequences from the African buffalo collected in 34 locations (treated as demes hereafter) across sub-Saharan Africa. First, we assumed an island model of connectivity between the demes and used the population pairwise *F*_ST_ matrix to estimate female migration rates through the formula 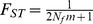 [24]. This was intended as an approximation only, as the equation only holds under certain assumptions–notably migration-drift equilibrium in Wright’s infinite island model–which are probably not met here [25]. However, it does allow us to explore the structure effect in a more complex and realistic scenario represented by unequal migration rates among demes. Simulations were carried out with the data-informed migration matrix and assuming a deme size of 500 females. We simulated data under a constant and a boom-bust demographic history. Replication in the data-informed simulations was slightly different from the idealised simulations because we now had to take into account that under a non-uniform migration matrix, it matters which populations are represented in the sample. Under *local* sampling we analysed ten replicates of each deme (340 data sets in total); under *pooled* sampling we analysed four samples from each of ten randomly selected demes, replicated 100 times. Under *scattered* sampling, we simply performed 100 replicates of the scenarios and analysed a single individual from each deme. Because of these differences in replication, the variance as evident from the different individual lines in the EBSPs is not directly comparable among the two parts of the analyses, as we discuss below.

### Skyline Plot Analyses

#### Simulated data analyses

All data sets were analysed with the extended Bayesian Skyline Plot (EBSP) coalescent prior in BEAST [2]. This model allows the data to guide the selection of the most probable piece-wise linear demographic function, hence in principle allows it to take any shape, although this is affected by prior settings. The number of change-points in the demographic function is influenced by the parameter ‘demographic.popSizeChange’ (PSC in the following), which was given a Poisson prior with a mean of ln(2). This corresponds to a prior assumption that zero and all non-zero PSCs have equal probability. For consistency and due to the low number of change points in the simple demographic scenarios explored here, we did not vary the PSC prior, but simulations have shown that it does influence the ability of EBSPs to pick up complex demographic patterns [12]. For each simulated scenario, we plotted the median inferred population size from each EBSP analysis (100 data sets per scenario) and the prior and posterior of the PSC parameter. The BEAST priors on substitution model parameters were chosen to conform to the values from the simulated data, and we fixed the substitution rate to that used in the simulations. BEAST analyses were run for 10^7^ steps drawing samples every 10^3^ step, which was found to be sufficient to reach convergence in trial runs.

To quantify the difference between simulated (i.e. the sum of all deme sizes) and inferred population sizes and supplement the visual observations of the EBSPs themselves, we calculated the following measures across all 100 simulation replicates for all constant-size simulations: Coverage, or the proportion of time points from the inferred EBSP demographic function where the 95% highest probability density interval included the simulated population size, and the mean (over all time points as above) relative departure (MRD) of the median inferred population size from the simulated one. These measures are related to those applied in Heled & Drummond [12].

#### Real data analyses

Finally, we applied the three different sampling strategies to the real D-loop data: *local* (all samples from each of the buffalo populations ranging from three to 85 individuals per population), *pooled* (four samples picked randomly from ten of the 34 populations, replicated 100 times) and *scattered* (one sample picked randomly from each population, replicated 100 times). It should be noted that this resampling is not equivalent to the replication of simulations to display coalescent stochasticity, since the case data represents just one realization of the coalescent process. It was done to ensure that results were not biased by sampling effects.

## Results

### Idealised Structured Population

**Figure 1:**
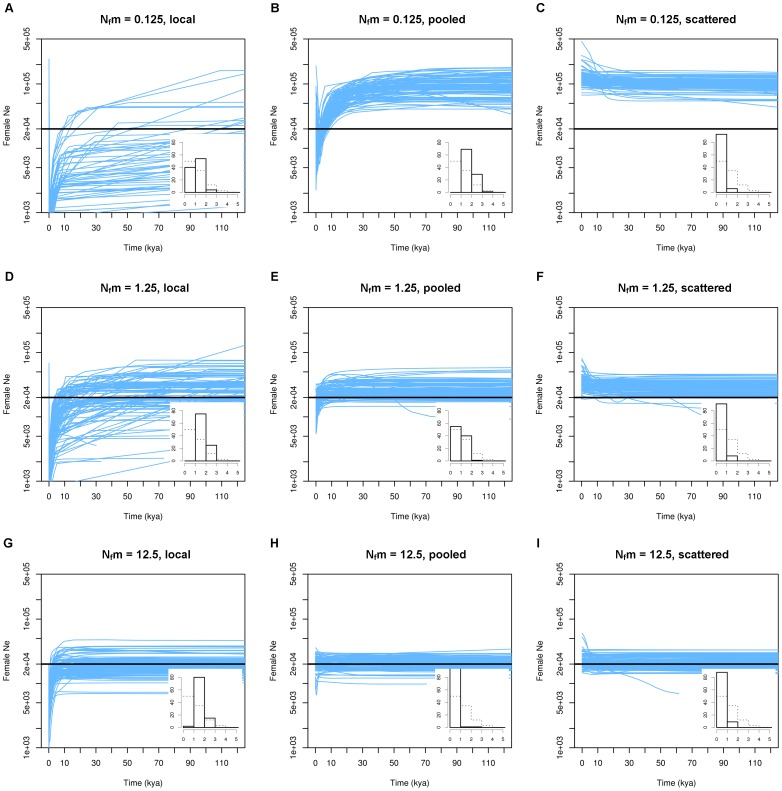
The structure effect in a 40-deme constant-size island model. For each scenario, 100 replicate data sets were generated and analysed with EBSPs. Light blue lines represent the median inferred female effective population size through time from each replicate. Time is measured in kya or thousands of years ago and is based on a molecular clock for buffalo D-loop sequences. Bold black lines represent the simulated size of the structured population (500 females * 40 demes = 20,000 females). Insert into each panel is a histogram of PSC values (on x-axis; see main text) across replicates. Dashed lines show the prior distribution for PSC. The y-axis in the insert histograms marks the frequency of occurrence in each PSC bin out of 100 replicates.

Our first series of simulations with no demographic change demonstrates that population structure can mimic population size changes in the absence of any such change (Fig. 1). The probability of such a misinterpretation depends on the interplay between the sampling strategy and the level of gene flow. *Locally* sampled scenarios under all levels of migration as well as *pooled* samples from the lowest migration class showed a clear trend towards declining EBSPs. The posterior distribution of the population size change (PSC) parameter confirmed this qualitative observation, as the above scenarios showed a strong tendency towards overestimating PSC (Fig. 1 insert panels). The measures of coverage and mean relative departure (MRD) corroborated the visual inspection of EBSPs and the PSC distributions, showing that the inferred population size at the present was notoriously lower than the simulated one under *local* sampling (Table 1). Other notable observations include a consistently higher inferred population size than the simulated one in the older parts of the EBSPs under *scattered* and *pooled* sampling for the two lowest migration classes (though less pronounced for the *pooled* strategy). This discrepancy between effective and actual (i.e total number of individuals) population size at low migration rates under *scattered* sampling is in fact expected as shown in Wakeley’s Eq. (6.18) [26]. The MRD of the inferred from the simulated population sizes is close to the expected magnitude of this effect (expected MRD from Wakeley’s Eq. (6.18): 4.00, 0.40 and 0.04 at *Nm* = 0.125, 1.25 and 12.5, respectively, to be compared with our calculated values of 4.33, 0.54 and 0.08; Table 1), showing the ability of EBSPs to correctly infer the structured effective population size in the collecting phase.

**Table 1:** Comparison of EBSP and simulated population sizes under different structural scenarios.

Scenario	*N_f_m*	EBSP	Sampling	coverage	MRD
constant	0.125	Fig. 1A	local	0.72	−0.77
island model	0.125	Fig. 1B	pooled	0.86	0.90
	0.125	Fig. 1C	scattered	0.61	4.33
	1.25	Fig. 1D	local	0.78	−0.76
	1.25	Fig. 1E	pooled	0.98	0.13
	1.25	Fig. 1F	scattered	0.90	0.54
	12.5	Fig. 1G	local	0.83	−0.50
	12.5	Fig. 1H	pooled	0.99	0.06
	12.5	Fig. 1I	scattered	0.99	0.08
10 demes	0.125	Fig. S1A	local	0.69	−0.70
	0.125	Fig. S1D	pooled	0.80	1.25
	1.25	Fig. S1B	local	0.77	−0.66
	1.25	Fig. S1E	pooled	0.97	0.31
	12.5	Fig. S1C	local	0.91	−0.30
	12.5	Fig. S1F	pooled	1.00	0.07
stepping stone	0.125	Fig. S2A	local	0.65	−0.77
	0.125	Fig. S2B	pooled	0.85	0.29
	0.125	Fig. S2C	scattered	0.61	7.01
	1.25	Fig. S2D	local	0.73	−0.76
	1.25	Fig. S2E	pooled	0.83	−0.33
	1.25	Fig. S2F	scattered	0.74	0.94
	12.5	Fig. S2G	local	0.76	−0.50
	12.5	Fig. S2H	pooled	0.90	−0.28
	12.5	Fig. S2I	scattered	0.99	0.14

Coverage is the proportion of time points at which the simulated population size lies within the inferred EBSP 95% highest probability density interval. MRD (mean relative departure) is the average relative deviation of the median inferred population size from the simulated size (e.g., 4.33 means that on average the inferred median population size is 4.33 times larger than the simulated one). For the 10 demes scenario, only *local* and *pooled* sampling was applied as *scattered* sampling would have resulted in low sample size (10 samples).

**Figure 2:**
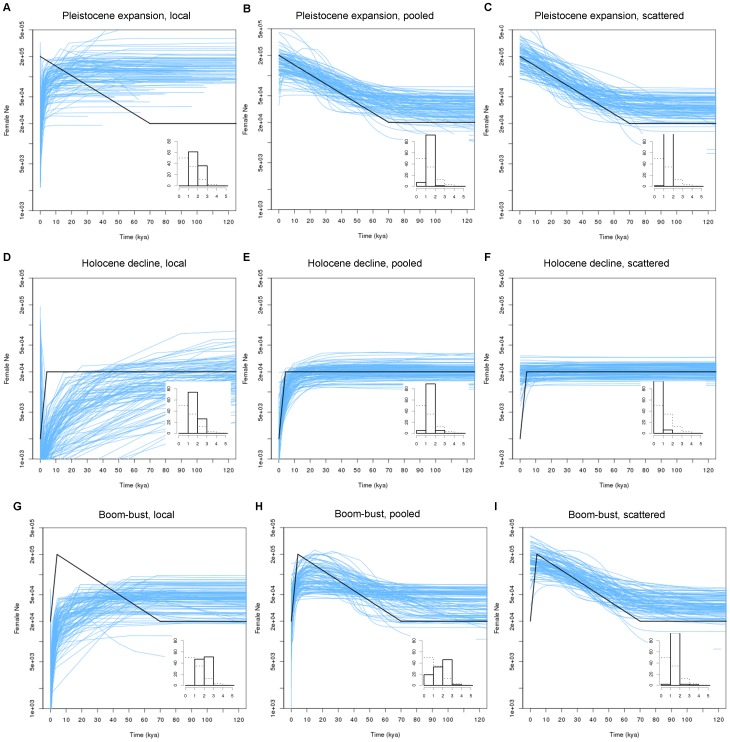
The structure effect in a 40-deme island model with demographic change. As Fig. 1, but the bold black line shows the simulated demographic change scenarios (see main text) with one or two changes in population size. Only the intermediary level of gene flow (N_f_m = 1.25) is shown.

When a single population change point was introduced (either a Holocene decline or a Pleistocene expansion) in the simulated demography we observed an interesting phenomenon. The expansion was unobservable in scenarios under *local* sampling (Fig. 2A), whereas it was the decline that was unobservable under *scattered* sampling (Fig. 2F). The *pooled* sampling strategy captured both phases more reliably (Fig. 2B,E). This pattern was also evident in the more complex boom-bust demographic simulations where both change points were included (Fig. 2G–I), although not all replicates of the *pooled* boom-bust scenario revealed the expansion phase (Fig. 2H).

The 10-deme scenarios showed the same overall trend as the 40-deme scenarios, but the severity of the structure effect was reduced relative to the comparable 40-deme scenarios (Fig. S1). Results for the stepping-stone simulations showed the same qualitative patterns as the island model simulations, but they were generally more extreme (Fig. S2). This is not surprising, as the stepping-stone model makes the effect of migration even more dominant over demographic changes because the average waiting time for lineages to find the same deme is longer. Results from the ‘IM-like’ models were similar to the permanent structure models (Table S1; Fig. S3).

### Data-informed Structured Population

**Figure 3:**
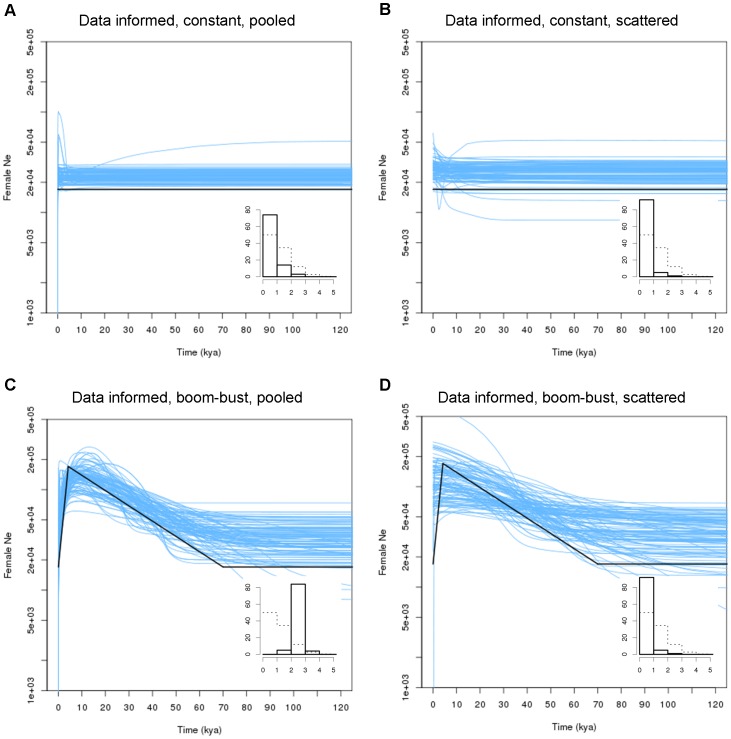
Two demographic scenarios under a real data informed island model. As Figs. 1 and 2, but the island model was modified to conform to the migration matrix estimated for a real biological system, the African buffalo. Only *pooled* and *scattered* sampling is shown. Bold lines mark the appropriate simulated population size. Replication was slightly different from Fig. 1 and 2 because it now matters which demes were included in the sample, see main text. Note also that the number of demes was 34, so the sum of the deme size differs from Fig. 1 and 2 (17,000 as opposed to 20,000 females).

Our simulations mimicking the inferred gene flow connecting 34 African buffalo populations revealed that under this structural configuration, the risk of a false signal of population size change was relatively low (Fig. 3) and resembled those of the high-migration scenarios under the idealised island model (Fig. 1G–I). Interestingly, the *local* sampling–when analysed deme by deme–revealed that there was a high correlation between the deme connectedness (measured as the mean of all pairwise *F*_ST_ values involving a given deme) and PSC (Fig. S4). This shows that within a structured population with unequal deme connectedness, the risk of false positives of population decline depends on which demes are sampled. We expanded on this observation by simulating *local* sampling in a structured population with a wide range of gene flow among demes (*N_f_m* 0.12–79.86, corresponding to an equilibrium *F*_ST_ of 0.007–0.827; see Supporting Information S1 for details). This revealed a clear separation between two phases in the relation between *F*_ST_ and PSC (which quantifies the risk of a structure effect): when *N_f_m* <2 (*F*_ST_ <0.2), there was a strong positive correlation between the two and when *N_f_m* >2, the correlation was negative (Fig. S4). The latter was initially surprising, but then we considered that in very isolated demes, there is a high probability that all lineages coalesce in the scattering phase (i.e. in the sampled deme) so that there will be no collecting phase (see the Discussion). We show EBSPs and PSC histograms for the two most extreme demes in terms of connectedness (Fig. S5) to illustrate the importance of deme connectedness.

**Figure 4:**
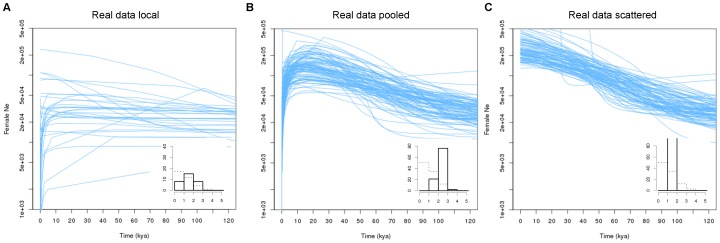
Three different sampling strategies for real data from the buffalo. *Local*, *pooled* and *scattered* sampling of real D-loop data from 34 African buffalo populations. The replication of each sampling strategy involved random drawing of the appropriate number of samples from demes as explained in the main text.

The African buffalo data yielded different results depending on the sampling strategy (Fig. 4). Under the *pooled* sampling we observed a conspicuous boom-bust signal that resembled the signal in the data-informed simulated data closely (Fig. 3F) and was very distinct from any of the false signals observed in Fig. 1. Under the *scattered* sampling the EBSP approached Fig. 2F and Fig. 3D, where the decline phase towards the present was almost absent. The qualitative differences between Fig. 4B and 4C were confirmed by the corresponding PSC values, with lower PSC values for the *scattered* sampling strategy (Fig. 4 insert panels). Finally, the locally sampled buffalo data showed variable skyline plots including boom-bust-like dynamics, pure expansions, pure declines and nearly constant population sizes (Fig. 4A). Note that low sample sizes for some of the demes (the three smallest samples consisted of three, four and ten individuals respectively) could lead to unreliable EBSPs.

## Discussion

### The Genealogical Background for the Structure Effect

Our results clearly demonstrate the dangers of using skyline plot methods for inferring demographic history without considering violations of the panmixia assumption. Overall, our results show that an apparent BSP population decline towards the present should always be regarded with caution, as it may be an artefact of structure. Such a confounding structure effect is not surprising, as it has been predicted in earlier theoretical studies [11], [14], [15], [17], [27] and identified in practical studies using various analysis methods [9], [10]. However, the effect has not been quantified under Bayesian skyline plot methods, which have become very popular in recent years. The intuitive, visual appeal coupled with a real risk of erroneous demographic inferences make BSPs vulnerable to misinterpretations. It should be underlined, however, that this is not a shortcoming of the methods themselves, but rather an under-appreciation of the dynamics of coalescent intervals in structured genealogies. As we show, the EBSPs actually do a fairly good job of inferring the theoretical effective size in the individual demes and the structured population (corresponding to the start of the scattering and the collecting phase, respectively). The problem is that these two are *expected* to differ (being smaller and larger than the sum of individuals across demes, respectively), even when the total population size remains constant over time. This phenomenon manifests itself as a declining population trajectory in BSPs. It should be noted that the structure effect in addition to the false positive of a population decline can also lead to the false negative of failing to detect a true population expansion towards the present, so the absence of a decline signal in a BSP is not necessarily evidence of the absence of a structure effect.

### Using Simulations to Assess the Risk of the Structure Effect

We show here that a critical approach is required before accepting the demographic history inferred from skyline plots. The inclusion of a case of real data allowed us to expand the scope of the analyses to include more complex connectivity among populations, to incorporate realistic historical events and to compare the idealised simulation results to analyses of real data, which we believe is a sound approach to validate BSP results in empirical studies [21]. Collectively, our simulations (not all of which we were able to report here) show that the magnitude of the structure effect depends on all of the following: the number of demes, the population size of the demes, the migration rate, the migration pattern (i.e. island or stepping-stone model, whether or not gene flow is equal among demes), the sampling scheme and the interaction between all of these. This makes it hard to predict the structure effect in a given biological system and *a priori* evaluate how it will affect demographic inferences. Hence, it is important to use simulations emulating biologically reasonable scenarios to evaluate whether an observed skyline plot is robust [7], [21]. When using the pairwise *F*_ST_ matrix of the D-loop data set to inform the migration matrix, we found evidence that *pooled* sampling should be able to distinguish between constant and fluctuating populations under this particular structural architecture, an important insight that was not evident from the idealised simulations. This part of the analyses also allowed us to make an important observation: the risk of a structure effect depends almost linearly on the ‘connectedness’ of the sampled demes up to a certain point (*F*_ST_ ∼ 0.2) beyond which it actually decreases slowly (Fig. S4), but at the cost of EBSPs no longer reflecting the size of the whole structured population, but rather that of the sampled deme only. These results are important for devising sampling strategies when the level of differentiation is approximately known.

The EBSPs from the real data closely resembled those from simulations including actual boom-bust dynamics, suggesting that we can explain the signal from the case data by invoking structure and demographic change according to the parameters of the data-informed simulations, but not by invoking structure alone. Although this does not prove that our case is entirely collapsible to the data-informed model with two demographic change points, it makes us more confident that such a model represents the demographic history of the buffalo reasonably well.

### Sampling Strategy and Practical Considerations

Locally collected samples always showed a false signal of population decline towards the present. Even at the high migration rate *N_f_m* = 12.5 (corresponding to an equilibrium *F*_ST_ of 0.04), *local* sampling resulted in a 97% false positive rate of mean PSC >1 in constant-size simulations (Fig. 1G). This is a very important observation underlining that *local* samples should not be used to draw conclusions about demographic history in the presence of even limited population structure. The reason for the false signals of population decline is conceptually simple. Looking backwards in time, the first part of the structured genealogy will be the scattering phase [14], [17] where the effective population size is estimated to something intermediary between that of the local deme and the structured population. The balance between these two is governed by the migration process; if all lineages coalesce within demes before any migration occurs, the inferred population size will correspond to the deme size, as was shown in the comparison of deme connectedness and PSC (Fig. S4). The structure effect is only present when there is a separation of the genealogy into a scattering and a collecting phase, so in very isolated demes the BSP will be more reflective of just the local deme population size (see also the low population size estimates and lower PSC in Fig. 1A and Fig. S2A). When the population enters the collecting phase, it starts behaving entirely like a structured population with an effective population size (1+1/*Nm*) times that of the census size (from equation 6.18 in [26]).

Although the *scattered* sampling approach does not suffer from the tendency to show false declines, this sampling strategy is apparently not optimal for inferring changes in the recent past (Fig. 2F). The reason for this is that under *scattered* sampling there is no scattering phase, so lineage migration has to take place before any coalescent events can occur. Unless the migration rate is very high (or the number of demes is very low) this leads to a low rate of coalescence in the recent part of the genealogy, hence reducing the inferential power in this period. This observation challenges the recommendation in [10] of using *scattered* sampling when inferring the recent demographic history of structured populations. We confirmed the robustness assertion, but found that power decreased concomitantly. In that study however, the object was explicitly to identify false positives of population size change (maximize robustness), and we confirm that this risk is lowest under *scattered* sampling.

On balance, the *pooled* strategy was the most appropriate sampling scheme under both the idealised and the data-informed structural architecture. This strategy was most capable of capturing both the expansion and the decline phase of the simulated population size change. It is of course important to emphasize that the *pooled* strategy can be varied continuously between the two extremes of *local* and *scattered* sampling and is hence less clearly defined than the two extremes. Consequently, the results presented here are strictly only valid for the somewhat arbitrarily chosen strategy of four samples from each of ten demes, which can be considered closer to the *scattered* than to the *local* extreme (any number *d* in the interval 2–20 samples could be sampled from any number between 2–20 demes to make reasonably balanced *pooled* sampling). As expected, changing *d* from 4 to 10 under the boom-bust scenario yielded a signal closer to that under *local* sampling (Fig. 2A), with the expansion phase becoming less detectable (results not shown). A *pooled* sampling strategy can thus be varied among the two extremes of *local* and *scattered* sampling according to the trade-off between the power to detect recent changes and the risk of getting a false positive from a structure effect.

Results from the three different sampling strategies applied to the real data confirmed that sampling can heavily influence the demographic signal in the presence of population structure. Therefore, we recommend that BSP-users at least explore different sampling schemes along the continuum between *local* and *scattered* sampling for their data sets, because it may reveal whether the structure effect is confounding the demographic inference. We recommend that whenever possible one considers the underlying population structure before planning sampling, yet in many cases some variety of *pooled* sampling will be desirable for inferring population dynamics if structure is present.

### Methodological Considerations

The coalescent effective population size is only defined for the collecting phase of a structured genealogy [14], [28], [29], therefore it was impossible to compare the inferred values with the expected ones throughout EBSPs. Consequently, our EBSP plots (Figs. 1, 2, 3, 4) and the measures calculated in Table 1 should not be regarded as an evaluation of the accuracy of EBSPs; rather they show the discrepancy between the sum of the deme sizes and the effective size in a structured population. However, the shape of the EBSPs and the PSC histograms demonstrate that even if EBSPs are accurate in inferring the theoretical (structured) effective population size at any given time, the change in effective size caused by the transition from a scattering to a collecting phase can be misleading if not interpreted in the proper context.

The replication of each scenario enabled us to assess the coalescent stochasticity [23] under any demographic scenario. As the plots show, individual EBSPs vary among repetitions–more so when the demographic history departs from simple, constant-sized populations (Fig. 2 and 3)–and only converge on the ‘true’ simulated demographic history when viewed across many repetitions (i.e. many independent loci). This demonstrates the importance of multi-locus data when inferring demographic history. The type of simulated marker (600bp of mtDNA) appeared suitable to shed light on the problem at hand, as the simulated demographics were always consistently detected in the EBSPs under at least one of the sampling strategies.

We stress that we were only able to address the structure effect on BSPs under a limited set of conditions. It is not possible to evaluate all the factors that influence the structure effect in a single study. Here we identify some general aspects of the problem, and we hope this will serve as a starting point for further studies on the factors that influence the structure effect in coalescent-based methods. As we show with the buffalo data, the structure effect will not always lead to serious misinterpretations, especially when a balanced (*pooled*) sampling strategy is followed.

The structure effect is not restricted to certain coalescent methods, but is rather a general problem that affects all methods that do not explicitly take subdivision into account. Hence, all methods that assume panmictic populations will suffer from confounding effects qualitatively similar to those reported here. One *ad hoc* approach to evaluate the structure effect in BSP analyses is to inspect the inferred genealogy and assess whether coalescent rates are obviously correlated with the structural conformation, i.e. if any substantial increase in coalescence rate towards the present predominantly occurs within demes. Ultimately, the best way of circumventing the confounding structure effect involves incorporating explicitly the spatial or structural information into the genealogical reconstruction. The LAMARC [30] and IM [31] software packages do this to some extent by allowing specification of population structure and hence co-estimation of migration and population size parameters, but currently they only handle simple demographic trajectories without any demographic change points [19], [20]. BSP methods could benefit from integration with such approaches. This should facilitate distinguishing between the transition from a scattering to a collecting phase and a true change in the census size of a structured population.

## Supporting Information

Figure S1 **The structure effect in a 10-deme population.** As Fig. 1, but simulating a 10-deme instead of a 40-deme population. Only local and pooled sampling was applied, as scattered sampling would have yielded unreasonably low sample sizes (10 samples).
(PDF)

Figure S2 **The structure effect in a 40-deme stepping-stone model.** As Fig. 1, but data were generated under a stepping-stone model (see main text). Notice change of y-axis scale in panel A.
(PDF)

Figure S3 **The structure effect in a population undergoing subdivision at various time points.** As Fig. 1, but scenarios constitute population subdivision rather than permanent structure. The time of the subdivision is: A–C: Last Glacial Maximum (LGM), 25,200 years ago; D–F: Mid-Holocene, 4200 years ago. Only local sampling was explored to reduce computation time.
(PDF)

Figure S4 **The correlation between connectedness and the risk of a structure effect.** Two sets of simulations (10 replicates) was carried out, one with 34 demes with mean pairwise *F*_ST_ of 0.007–0.123 and one with mean pairwise *F*_ST_ of 0.069–0.827. *Local* sampling was performed and EBSPs and PSC values were obtained. A linear regression of mean PSC on mean pairwise *F*_ST_ was done separately for each set. See main text and for corresponding values of *N_f_m*.
(PDF)

Figure S5 **EBSPs of the two most extreme demes in the connectedness analysis.** Ten replicates was performed for each deme. Here we show EBSPs of the most (A) and least (B) ‘connected’ demes, respectively (characterised by a mean pairwise *F*_ST_ of 0.007 and 0.827, respectively). See Fig. S4 and caption.
(PDF)

File S1 **BSSC example input file corresponding to the simulation depicted in** **Fig. 1A** **.**
(PAR)

File S2 **BSSC example input file corresponding to the simulation depicted in** **Fig. 2H** **.**
(PAR)

Supporting Information S1 **Additional information about various analyses as referenced in the main text.**
(DOCX)

Table S1 **As** **Table 1** **; ‘IM-like’ scenarios (see Supporting Information S1).** Only *local* sampling was explored.
(DOCX)
