# Supplementary figures and images for: The Confounding Effect of Population Structure on Bayesian Skyline Plot Inferences of Demographic History
Source: PLoS One. 2013 May 7;8(5):e62992. doi: 10.1371/journal.pone.0062992 (PMC3646956; doi:10.1371/journal.pone.0062992)

**A****LGM,  $N_f m = 0.125$** 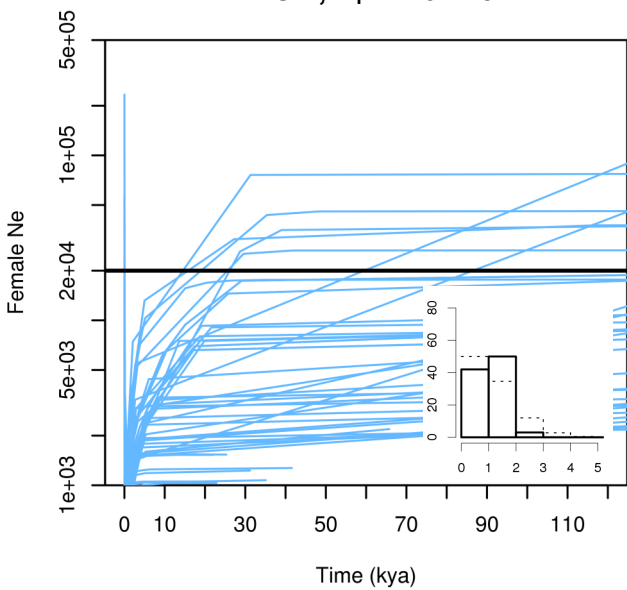**B****LGM,  $N_f m = 1.25$** 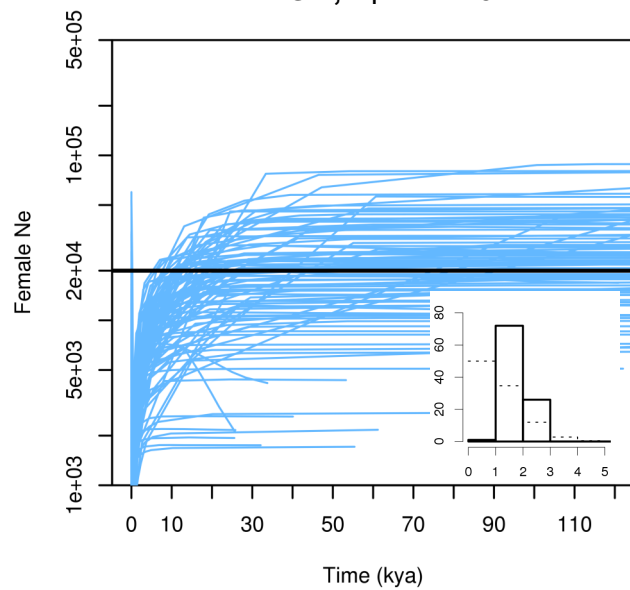**C****LGM,  $N_f m = 12.5$** 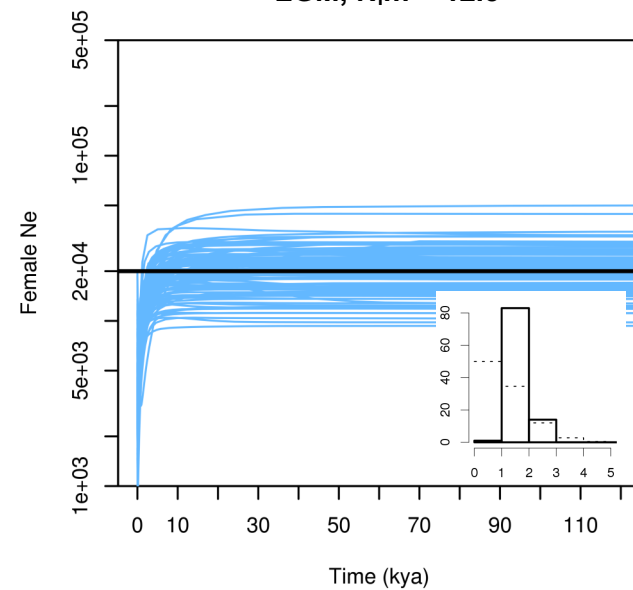**D****Holocene,  $N_f m = 0.125$** 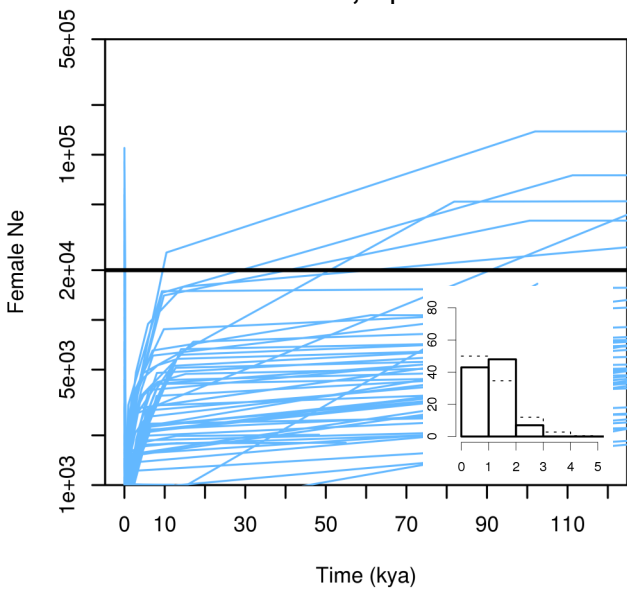**E****Holocene,  $N_f m = 1.25$** 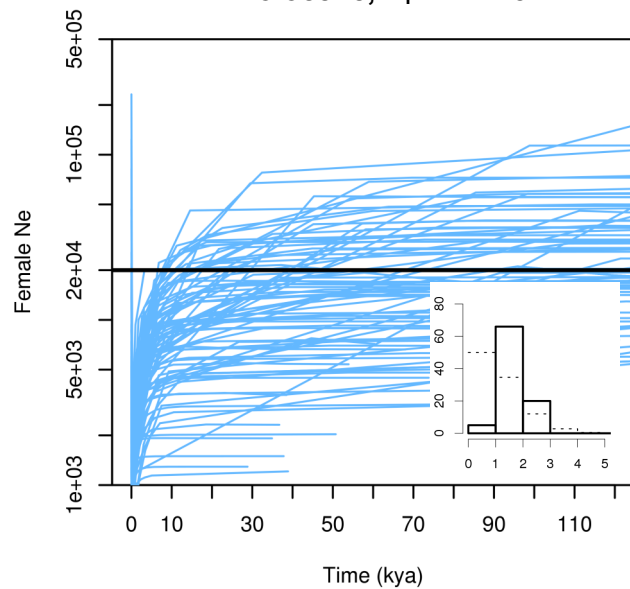**F****Holocene,  $N_f m = 12.5$** 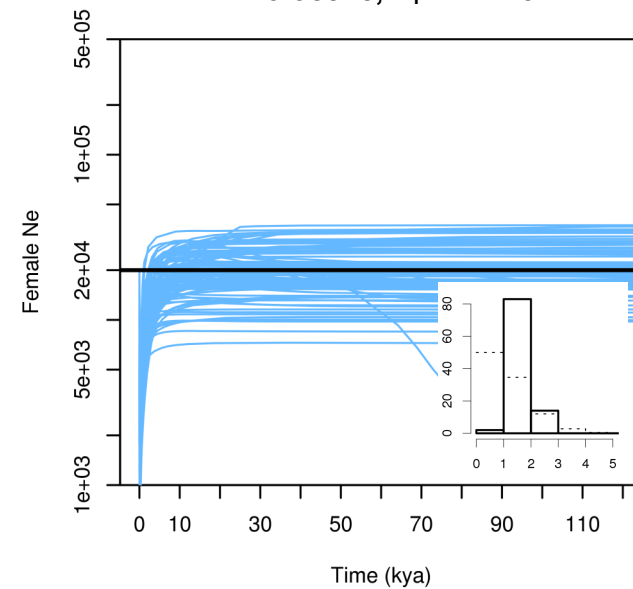

Supplement: Figure S1 — The structure effect in a 10-deme population. As Fig. 1, but simulating a 10-deme instead of a 40-deme population. Only local and pooled sampling was applied, as scattered sampling would have yielded unreasonably low sample sizes (10 samples). (PDF) [file pone.0062992.s001.pdf]

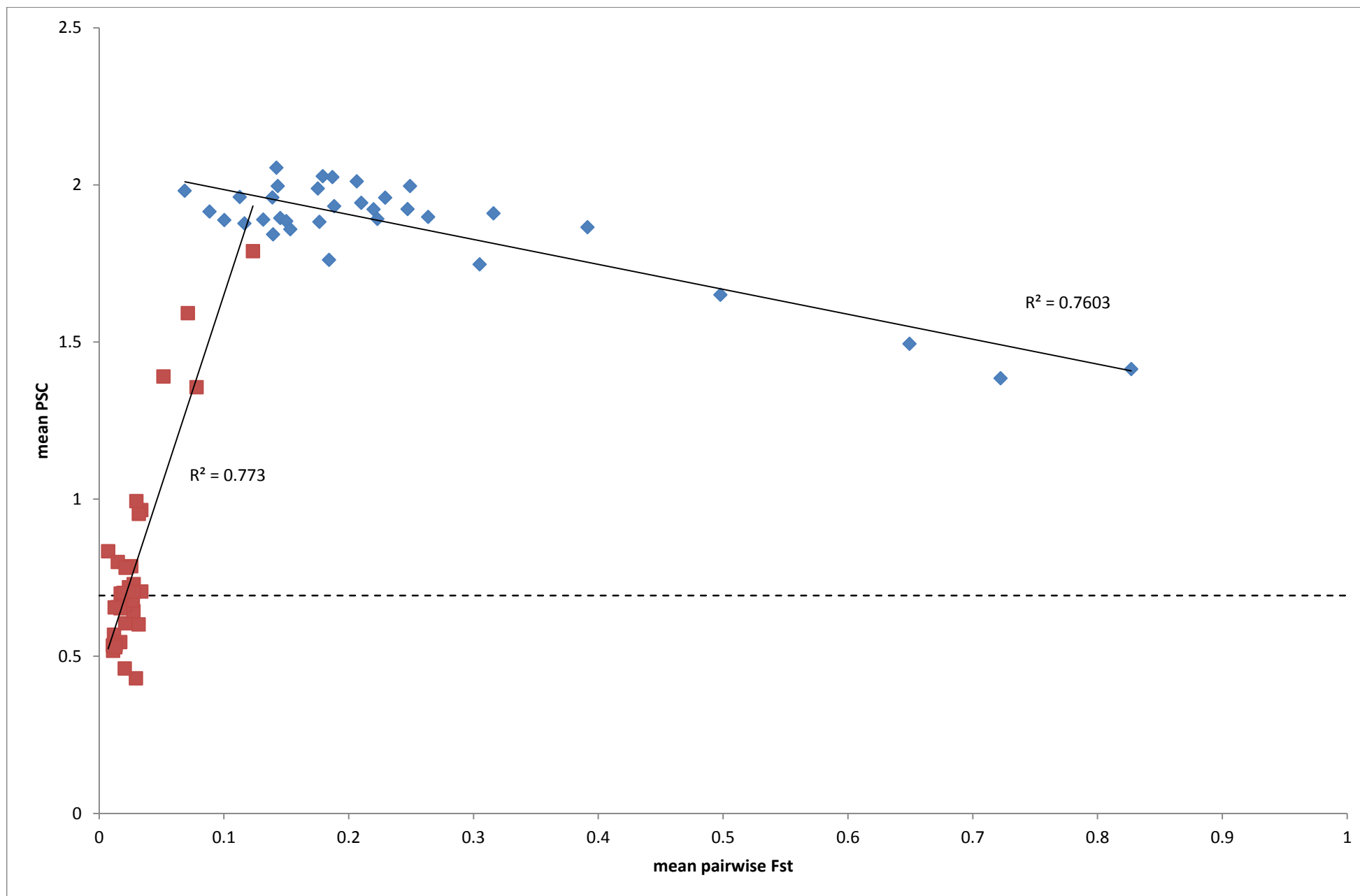

Supplement: Figure S4 — The correlation between connectedness and the risk of a structure effect. Two sets of simulations (10 replicates) was carried out, one with 34 demes with mean pairwise F ST of 0.007–0.123 and one with mean pairwise F ST of 0.069–0.827. Local sampling was performed and EBSPs and PSC values were obtained. A linear regression of mean PSC on mean pairwise F ST was done separately for each set. See main text and for corresponding values of Nfm. (PDF) [file pone.0062992.s004.pdf]

$F_{ST} = 0.827$ , local

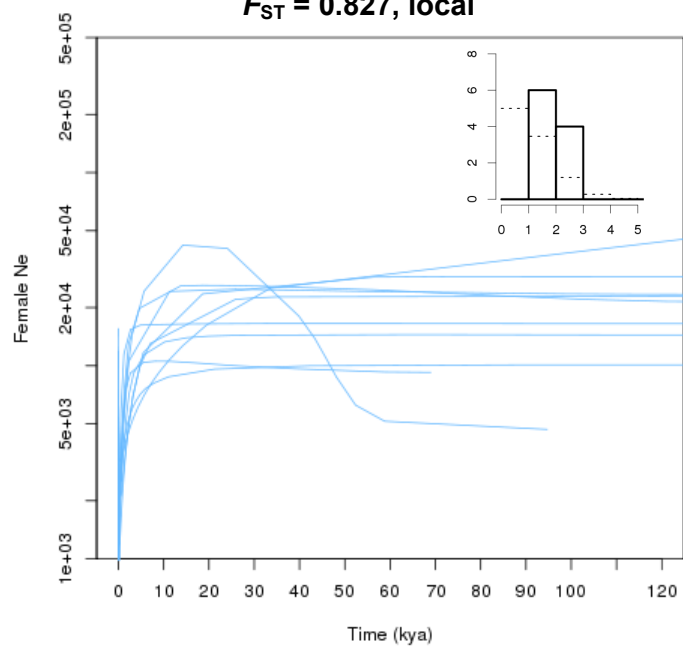

$F_{ST} = 0.007$ , local

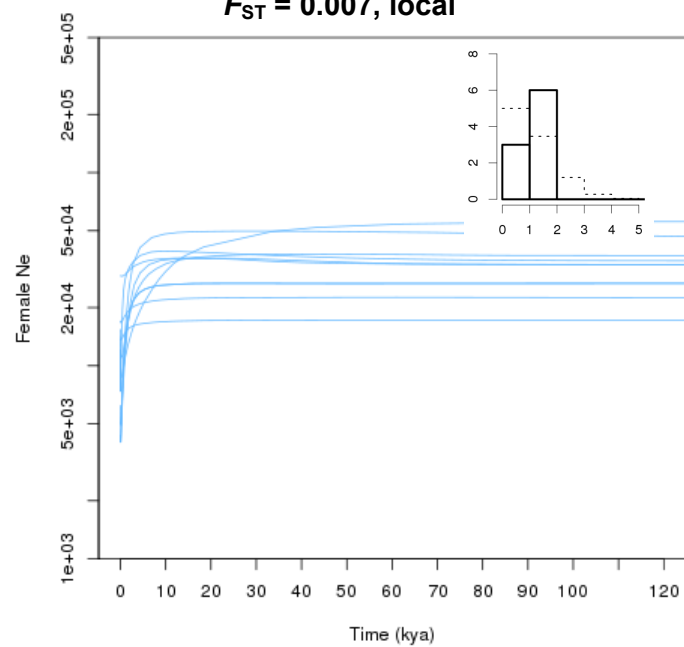

Supplement: Figure S5 — EBSPs of the two most extreme demes in the connectedness analysis. Ten replicates was performed for each deme. Here we show EBSPs of the most (A) and least (B) ‘connected’ demes, respectively (characterised by a mean pairwise F ST of 0.007 and 0.827, respectively). See Fig. S4 and caption. (PDF) [file pone.0062992.s005.pdf]
