# Supplementary material for: The Confounding Effect of Population Structure on Bayesian Skyline Plot Inferences of Demographic History
Source: PLoS One. 2013 May 7;8(5):e62992. doi: 10.1371/journal.pone.0062992 (PMC3646956; doi:10.1371/journal.pone.0062992)
Supplement: Figure S2 — The structure effect in a 40-deme stepping-stone model. As Fig. 1, but data were generated under a stepping-stone model (see main text). Notice change of y-axis scale in panel A. (PDF) [file pone.0062992.s002.pdf]

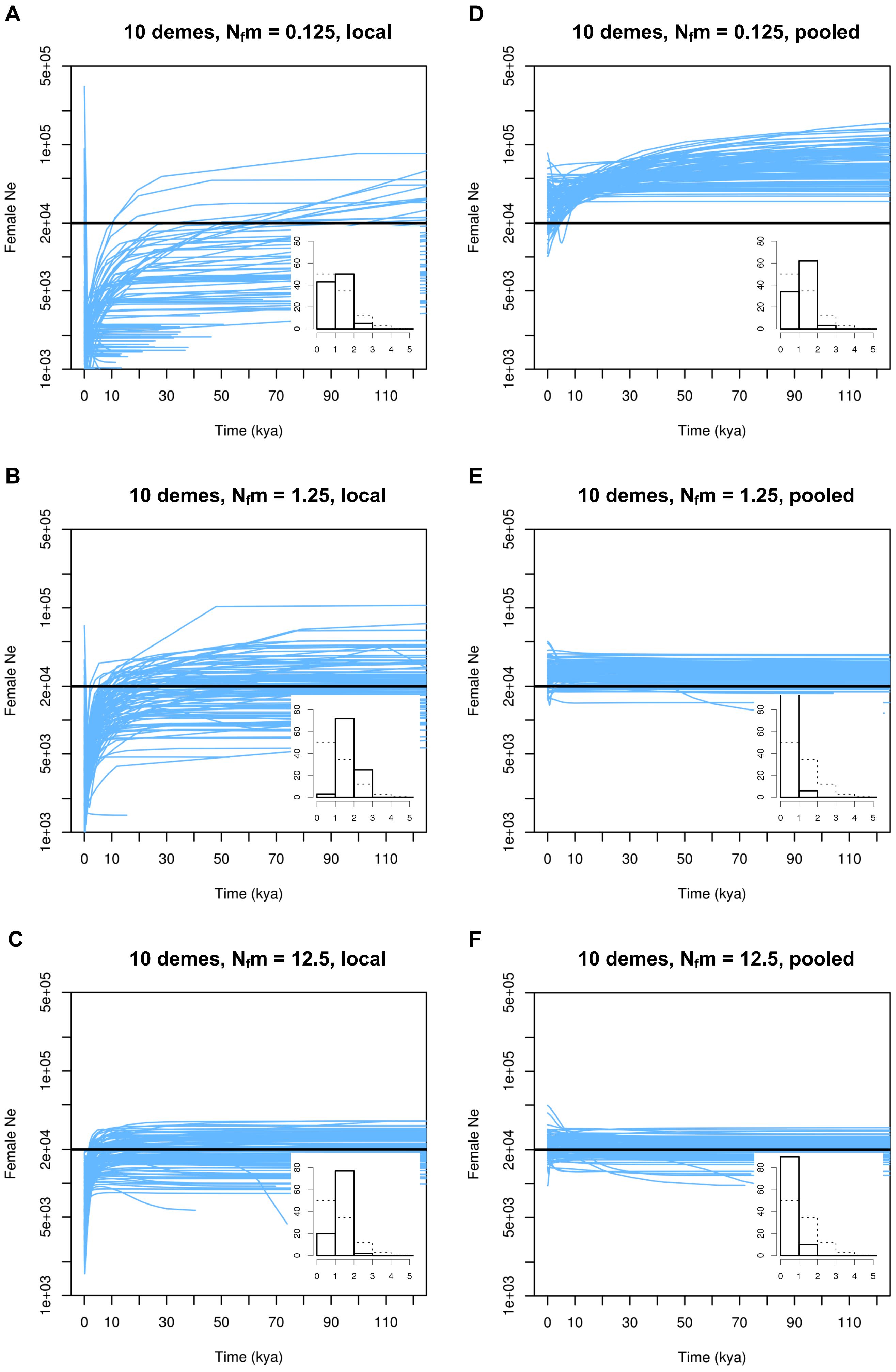

A
10 demes, $N_f m$ = 0.125, local
D
10 demes, $N_f m$ = 0.125, pooled
B
10 demes, $N_f m$ = 1.25, local
E
10 demes, $N_f m$ = 1.25, pooled
C
10 demes, $N_f m$ = 12.5, local
F
10 demes, $N_f m$ = 12.5, pooled
Female Ne
1e+03
5e+03
2e+04
1e+05
5e+05
Time (kya)
0
10
30
50
70
90
110
0 1 2 3 4 5
0 20 40 60 80
