# Supplementary material for: The Confounding Effect of Population Structure on Bayesian Skyline Plot Inferences of Demographic History
Source: PLoS One. 2013 May 7;8(5):e62992. doi: 10.1371/journal.pone.0062992 (PMC3646956; doi:10.1371/journal.pone.0062992)
Supplement: Figure S3 — The structure effect in a population undergoing subdivision at various time points. As Fig. 1, but scenarios constitute population subdivision rather than permanent structure. The time of the subdivision is: A–C: Last Glacial Maximum (LGM), 25,200 years ago; D–F: Mid-Holocene, 4200 years ago. Only local sampling was explored to reduce computation time. (PDF) [file pone.0062992.s003.pdf]

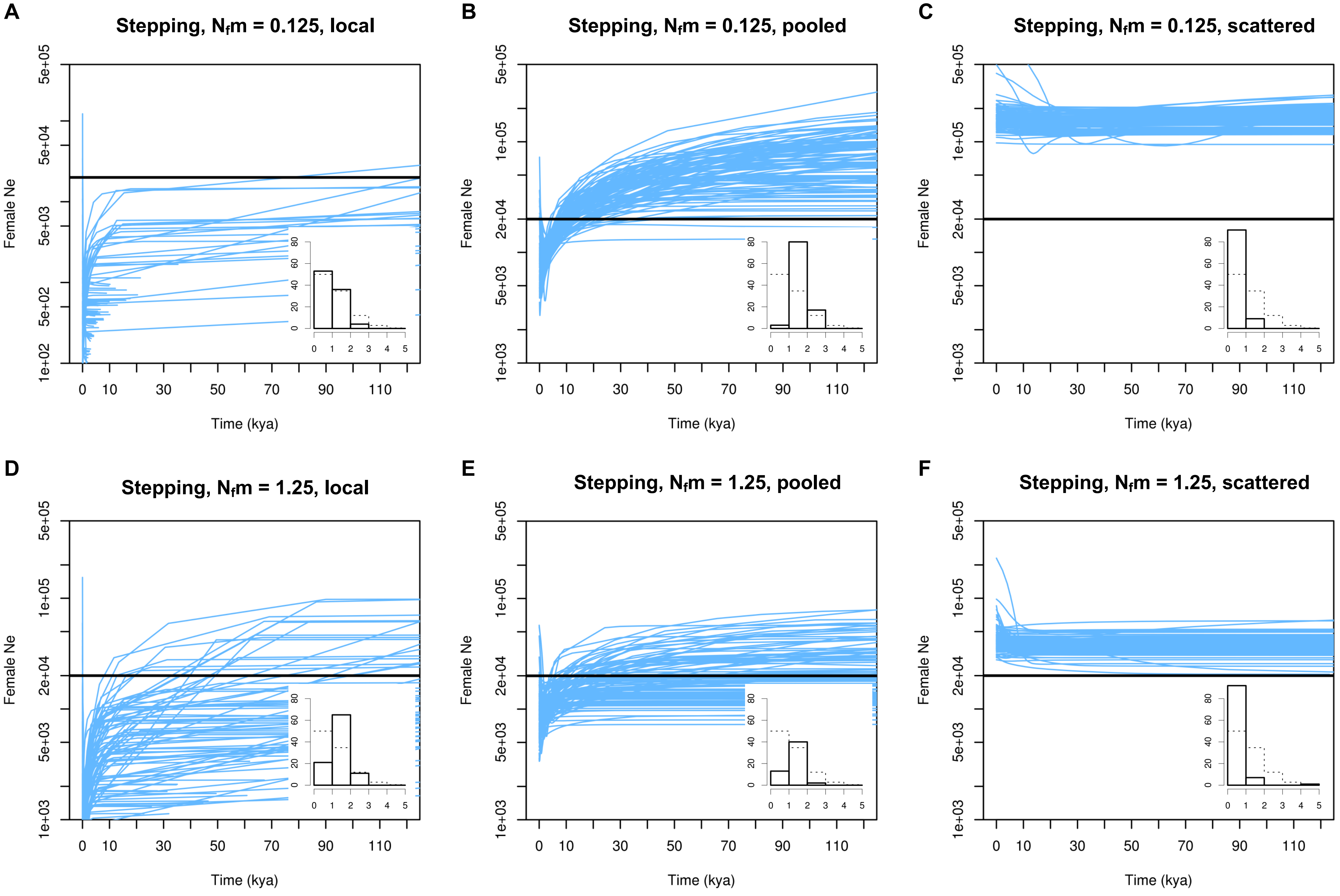
A
Stepping, $N_fm$ = 0.125, local
B
Stepping, $N_fm$ = 0.125, pooled
C
Stepping, $N_fm$ = 0.125, scattered
D
Stepping, $N_fm$ = 1.25, local
E
Stepping, $N_fm$ = 1.25, pooled
F
Stepping, $N_fm$ = 1.25, scattered
Female Ne
Time (kya)
1e+02
5e+02
5e+03
5e+04
5e+05
1e+03
5e+03
2e+04
1e+05
0
10
30
50
70
90
110
0
20
40
60
80
1
2
3
4
5

Figure S1

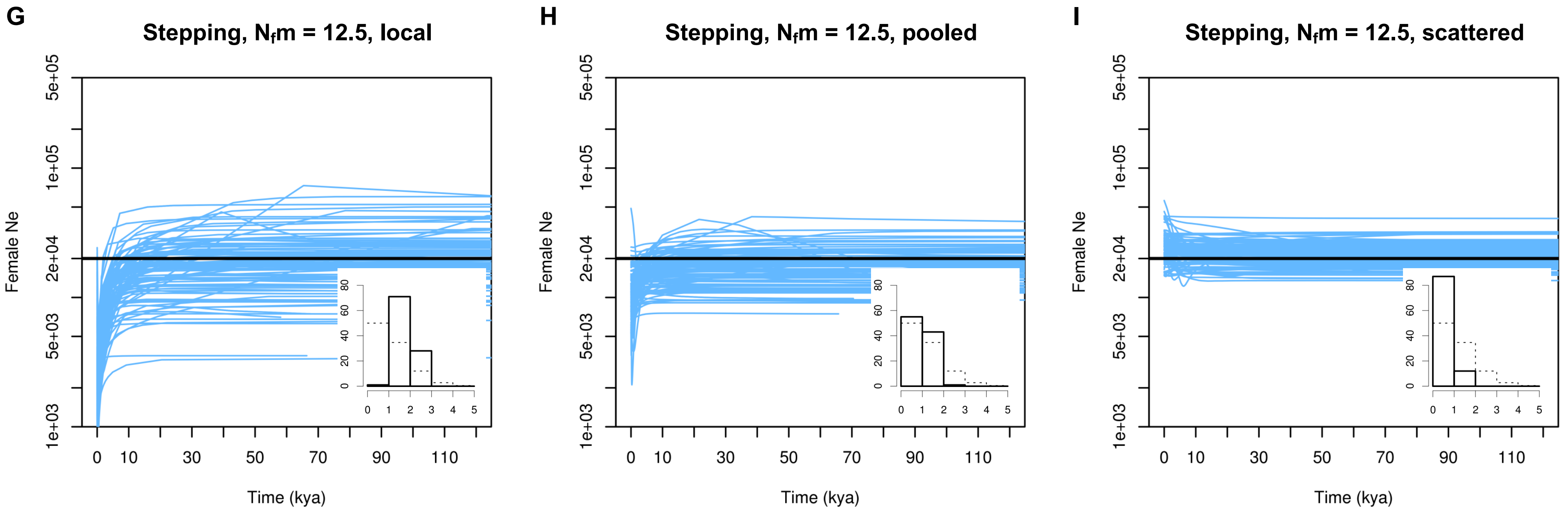


Figure S1 cont.
