# Supplementary material for: The Confounding Effect of Population Structure on Bayesian Skyline Plot Inferences of Demographic History
Source: PLoS One. 2013 May 7;8(5):e62992. doi: 10.1371/journal.pone.0062992 (PMC3646956; doi:10.1371/journal.pone.0062992)
Supplement: Supporting Information S1 — Additional information about various analyses as referenced in the main text. (DOCX) [file pone.0062992.s008.docx]

**Supporting Information, main document**

***Scenarios with changes in population size or structure***

To extend our results beyond the most simple, stationary scenarios with permanent structure, we incorporated demographic change at various key time points in a series of more complex scenarios. These involved changes in the population size (a single change point or two change points, respectively) or changes in the structural conformation (‘IM-like models, see below). The time points were chosen to comply with important events in the demographic history of the case species, the African buffalo: 1) mid-Holocene (4200 years or 600 buffalo generations ago); 2) Last Glacial Maximum (25,200 years or 3600 buffalo generations ago); 3) the late Pleistocene (70,000 years or 10,000 buffalo generations ago). However, we believe the time points are relevant for a range of megafauna species (Lorenzen et al. 2012), as they have been identified as important events in the late Pleistocene and the Holocene. Note that the absolute times are associated with the assumed buffalo generation time, and that they would change proportionally with a different generation time.

In the population size change scenarios, the population size started at ancestral N_f_ = 20,000 (summed over demes) and decreased/expanded ten-fold over the relevant time period. In the boom-bust scenarios, the population first expanded ten-fold and then decreased ten-fold.

***Additional scenario settings***

*Stepping-stone model*

In the stepping-stone model we applied the same overall migration rates as in the island model, but now demes were only exchanging migrants with a subset of the remaining demes. The deme network consisted of a two-dimensional 10 x 4 rectangular lattice without periodic boundary conditions, meaning that edge demes had fewer connections than central demes.

*‘IM-like’ models*

To explore the structure effect under non-permanent structure, we simulated data under scenarios of population subdivision from a previously panmictic population (‘IM-like’ conditions [Hey & Nielsen REF]) at two different times as described above: 1) the onset of the Last Glacial Maximum (25,200 years ago; 3600 buffalo generations) and 2) the mid-Holocene (4200 years ago; 600 buffalo generations). Otherwise conditions were identical to the idealized scenarios: population size was maintained constant at 500 females per deme in 40 demes, and gene flow was as described in the main text. The ‘IM-like’ scenarios showed similar results to the permanent-structure, constant-size scenarios (Table S1; Fig. S3). Although results were similar across different time points of the onset of subdivision, there was a tendency that the intermediate-age (Holocene) subdivision scenario caused slightly higher relative departure of the inferred population size than recent and older subdivision scenarios (Table S1).

***Connectedness and PSC analysis***

We simulated data under a 40-deme island model with unequal gene flow corresponding to equilibrium pairwise *F*_ST_ values of 0.007–0.827. The simulations were performed in two sets, one with gene flow corresponding to pairwise *F*_ST_ of 0.007–0.123 (Nm: 4.18–79.86), the other to pairwise *F*_ST_ of 0.069–0.827 (Nm: 0.12–7.98). Each deme was sampled locally and analyzed using EBSPs as described in the main text. We calculated the mean inferred PSC for each deme across ten replicates and plotted it against the mean pairwise *F*_ST_ to see the correlation between deme connectedness and the risk of a structure effect. Note that the demes do not strictly represent independent data points, as they were interconnected with the other demes in the simulation.

***Additional files***

We supply two example .par files, the input for the BSSC simulation program:

- File S1: Idealised_local_constant_Nfm0.125: corresponding to results shown in Fig. 1A.
- File S2: Idealised_pooled_boombust.par: corresponding to results shown in Fig. 2H.
