# Supplementary material for: The Confounding Effect of Population Structure on Bayesian Skyline Plot Inferences of Demographic History
Source: PLoS One. 2013 May 7;8(5):e62992. doi: 10.1371/journal.pone.0062992 (PMC3646956; doi:10.1371/journal.pone.0062992)
Supplement: Table S1 — As Table 1 ; ‘IM-like’ scenarios (see Supporting Information S1). Only local sampling was explored. (DOCX) [file pone.0062992.s009.docx]

| **Scenario** | ***N_f_m*** | | **EBSP** | | **Sampling** | | **coverage** | | **MRD** | |
| --- | --- | --- | --- | --- | --- | --- | --- | --- | --- | --- |
| LGM | | 0.125 | | Fig. S3A | | local | | 0.69 | | -0.75 |
|  | | 1.25 | | Fig. S3B | | local | | 0.76 | | -0.78 |
|  | | 12.5 | | Fig. S3C | | local | | 0.84 | | -0.48 |
|  | |  | |  | |  | |  | |  |
| holocene | | 0.125 | | Fig. S3D | | local | | 0.71 | | -0.82 |
|  | | 1.25 | | Fig. S3E | | local | | 0.76 | | -0.82 |
|  | | 12.5 | | Fig. S3F | | local | | 0.82 | | -0.53 |
|  | |  | |  | |  | |  | |  |
| rinderpest | | 0.125 | | Fig. S3G | | local | | 0.73 | | -0.66 |
|  | | 1.25 | | Fig. S3H | | local | | 0.79 | | -0.75 |
|  | | 12.5 | | Fig. S3I | | local | | 0.83 | | -0.53 |
